# Utilization of Infrared Drying as Alternative to Spray- and Freeze-Drying for Low Energy Consumption in the Production of Powdered Gelatin

**DOI:** 10.3390/gels10080522

**Published:** 2024-08-09

**Authors:** Ümran Cansu

**Affiliations:** Organized Industrial Zone Vocational School, Harran University, 63200 Şanlıurfa, Turkey; umrancansu@harran.edu.tr

**Keywords:** fish skin-derived gelatin, infrared drying, spray-drying, freeze-drying, energy consumption, gel strength

## Abstract

This study evaluated possible utilization of infrared drying (ID) as an alternative to spray- (SD) and freeze-drying (FD) for fish skin-derived gelatins. Physical, functional, thermal, and spectroscopic analyses were conducted for characterization of the resulting gelatin powders. Energy consumption for the applied drying methods were 3.41, 8.46 and 25.33 kWh/kg for ID, SD and FD respectively, indicating that ID had the lowest energy consumption among the studied methods. Gel strength, on the other hand, was lower (398.4 g) in infrared-dried gelatin (ID-FG) compared to that (454.9 g) of freeze-dried gelatin (FD-FG) and that (472.7 g) of spray-dried gelatin (SD-FG). TGA curves indicated that ID-FG showed more resilience to thermal degradation. SDS-PAGE and UV-Vis spectra indicated that slight degradation was observed in the β-configuration of ID-FG. ID-FG and SD-FG gelatins had the highest water holding capacity (WHC), protein solubility and transparency values compared to that of FD-FG. Morphological structures of the samples were quite different as shown by SEM visuals. Ultimately, the findings showed that infrared drying may be a promising alternative for gelatin processing, maintaining product quality and supporting sustainable practices in food and other industries.

## 1. Introduction

Gelatin obtained from the thermal denaturation of collagen that is a structural protein within the connective tissues of animals is evaluated for various applications including food, pharmaceuticals, and cosmetics [1,2]. The growing demand for gelatin has prompted an in-depth evaluation of fish processing residues as a viable alternative source. Therefore, with increases in fish processing residues, fish gelatin production has increased and become a sustainable and more environmentally friendly alternative [2,3,4].

Gelatin production comprises several essential stages: initial pre-treatments involving alkali and acid treatments, followed by extraction and subsequent drying processes. The quality and functionality of powder gelatin are influenced significantly by different factors, for instance, the choice of raw materials, pre-treatments, extraction conditions, and the techniques utilized for drying [5,6]. Among these, the drying process is a critical step in the production of gelatin powders. During drying, covalent and non-covalent bonds in gelatins are broken, resulting in alterations or modifications in the structure. These changes significantly alter the quality, physical and chemical attributes namely gel strength, viscosity, solubility, water and fat absorption ability, foaming and emulsification properties of gelatins [5,7].

Various methods such as air-, freeze-, and spray-drying have remarkable impacts on the characteristics of gelatin molecules [6,8]. Feng et al. [6] reported that spray-drying reduces the degradation of gelatin, retains a greater number of α chains, and decreases solubility. Furthermore, this method significantly enhances the emulsifying properties of gelatin due to the increased presence of gelatin particles. These improvements are more pronounced when compared to the outcomes of freeze-drying and hot air drying. Salem et al. [8] found that the freeze-drying and spray-drying influenced the properties of dogfish skin gelatin gels by altering their structure. Notably, spray-drying enhanced functional properties, including water holding capacity, fat binding capacity, and foaming properties. Kanwate et al. [7] demonstrated that spray-drying, despite yielding a lower output, significantly enhances both the gel properties and the functional characteristics of gelatins in comparison to freeze-drying and vacuum drying methods. Spray-drying is extensively employed in the food industry owing to its cost-effectiveness, efficiency in time, and ability to produce high-quality powder. Moreover, this method leads to the reduction of undesirable odors in fish skin gelatin [9]. Nevertheless, the spray-drying process tends to result in greater particle shrinkage and denser particles. In contrast, gelatins powdered by freeze-drying maintain their native structural integrity, as they experience fewer stresses related to thermal and water evaporation. However, the institutional costs and the amount of energy consumption during spray- and freeze-drying are quite high [10].

Novel drying approaches such as infrared drying, ultrasound-assisted drying and hybrid-drying (e.g., hot air–infrared, infrared–vacuum, microwave–infrared and microwave–vacuum drying) have become ubiquitous in the food industry [11]. Infrared radiation (IR) is a component of the electromagnetic spectrum and acts like the heating effect of the sun. During the use of IR to dry or heat a material, the surface layer of solid material absorbs IR. However, radiation goes through to some extent in moist, porous materials; their capability to transmit depends on the moisture content [12,13]. Infrared heat drying has become a common technique in food applications due to its low drying time, low installation cost, the high quality of the final product, and better energy savings [13].

However, there is no information in the literature regarding the impact of infrared drying on physicochemical and functional properties of gelatin derived from fish skin and drying energy consumption values. Therefore, this study aimed to incorporate infrared drying technique into the gelatin production process. For this objective, gelatin solutions obtained from fish skin were dried by infrared-, spray- and freeze-drying methods. Energy consumption values of different drying methods were calculated. Also, the quality, functional, thermal and spectroscopic characteristics of powdered fish gelatin were investigated.

## 2. Results and Discussion

### 2.1. Effect of Different Drying Procedures on the Physical Features of Gelatin

Different drying methods caused quite remarkable changes in the functional properties of fish gelatin. WHC and FBA, which are among the basic functional properties of gelatin, represent its ability to trap water and oil between molecules. Among the samples, significant differences were determined in WHC values, and SD-FG was considerably higher in WHC (Table 1). This was probably due to the extent of hydrophilic residues that occurred during spray-drying [14]. FD-FG samples were significantly (*p* < 0.05) higher in terms of FBA (15.39 ± 0.2) compared to that of others, which might be caused by higher amounts of hydrophobic residues, such that presented observations strongly suggest that higher FBA also corresponds with lower WHC [15,16].

Gelatin solubility is a crucial feature for its use in food systems. The ID-FG (96.36 ± 3.6) and SD-FG (96.55 ± 2.5) samples presented higher solubility than FD-FG (90.15 ± 3.1). Similarly, Sae-Leaw et al. [10] mentioned that the spray-dried seabass skin gelatin performed with greater solubility compared to freeze-dried gelatin. However, Kanwate et al. [7] reported the freeze-dried gelatin from the swim bladder of *Labeo rohita* exhibited slightly higher solubility than spray-dried gelatin. The difference in solubility ratio could be related to the amount of subunits of gelatin and hydrophobicity of it. Also, this difference can be attributed to the effects of drying methods on the molecular weight of peptides and the relative content of polar and non-polar groups within the amino acid composition [17]. Carr index and Hausner ratio are utilized as metrics for assessing the flow properties and cohesiveness of powder products. Generally, a Carr index exceeding 25 and/or a Hausner ratio exceeding 1.35 indicate poor flowability of a powder [18]. Different drying methods significantly affected the powder properties. The ID-FG samples exhibited good to excellent flow properties, with a Carr index of 25.26 ± 1.50 and a Hausner ratio of 1.34 ± 0.03.

The transparency of fish gelatin solutions was associated with impurities such as oil, raw material particles, etc. in the solution [4]. The SD-FG gelatin sample formed a statistically much more transparent (53.00 ± 0.4) solution than the others. Additionally, the ID-FG sample exhibited more transparent solutions than the freeze-dried samples. It seems clear that the thermal processes applied in drying were found to be very efficient on the transparency of gelatin. According to the obtained results, it was determined that heat treatment during drying produces a more transparent gelatin solution. This transparency could be attributed to the thermal treatment’s ability to retain impurities. However, the lower solubility of the FD-FG powder may also influence the transparency ratio. Inadequate washing stages following acid pretreatment and mineral substances that pass into the solution from the fish skin affected the pH value of the gelatin, which plays a crucial role in food application [4,16]. However, pH and conductivity values did not change in the gelatins obtained from different drying methods.

The color attribute does not have any effect on functional features of gelatin, but it has an effect on consumer acceptability. SD-FG powder displayed a higher L*-value than those of other dried gelatins (*p* < 0.05). The elevated L*-values observed in spray-dried gelatin powders can be attributed to the specific drying mechanism employed and the limited duration of exposure to temperature [10]. The chroma value, which reflects the intensity of color saturation and shows a positive correlation with b*-value, was found as a 30.70 ± 0.8, 7.32 ± 0.7 and 14.85 ± 1.1 for ID-FG, SP-FG and FD-FG, respectively (*p* < 0.05). The hue angle correlates with changes in the a* and b* values, where a hue angle of 90° corresponds to pure yellow and a hue angle of 0° corresponds to pure red [10,19]. During infrared drying at elevated temperatures, non-enzymatic browning reactions may occur to varying extents. This was also evidenced by a more pronounced yellowish hue. 

### 2.2. Electrical and Specific Energy Consumption of Different Drying Procedures

In developed countries, the energy expended on heat during the drying process constitutes approximately 12 to 40% of the total industrial energy consumption. This energy usage accounts for 20 to 70% of the overall production costs, depending on the type of industry. Therefore, all sectors aim to reduce the costs of stages such as drying [20,21]. Table 2 shows the electricity consumption values calculated for three different drying methods applied to gelatin. It was determined that the infrared dryer exhibited the lowest electricity consumption (0.217 ± 0.00 kW*h/g gelatin). Additionally, infrared drying was identified as the fastest method for drying of gelatin extracts (45.96 ± 0.11 min/g gelatin). This was probably due to rapid heating of gelatin extracts by infrared (IR) radiation. The higher the heat density and the deeper the penetration, the greater the rate of moisture migration toward the surface [21,22]. Previous studies have already reported that infrared drying exhibited a shorter time regarding the drying process, meaning less electrical energy is required in drying plants [23,24].

The results showed that specific energy consumption was significantly (*p* < 0.05) affected by drying methods in gelatin extract. Energy consumption for ID-FG, SD-FG and FD-FG was 3.41, 8.46 and 25.33 kW*hkg, respectively. In drying gelatin extract, the lowest energy consumption was related to the infrared method. The infrared drying method reduced drying time due to the effective high heat density, which accelerated moisture removal from the extract surface. Consequently, as drying time decreased, the total energy consumption also decreased [24]. The longer time spent on drying in the FD method for the gelatin extract resulted in the highest energy consumption. Similar results were mentioned by Kaveh et al. [24] and Xu et al. [25]. Based on the energy consumption values, it has been conclusively determined that infrared drying is the most effective method for drying gelatin extracts.

Infrared (IR) drying operates by transferring energy directly to the material without heating the surrounding air or requiring an intermediary heating medium between the energy source and the material. This direct energy transfer facilitates rapid and uniform heating, as IR radiation penetrates directly into the material’s internal layers. Therefore, the energy efficiency of infrared drying is closely linked to the material’s absorption characteristics. Furthermore, several other factors play a crucial role in determining energy efficiency, including the distance of the heating source and the material surface, the velocity and temperature of the airflow, and the thickness of the material layer. Each of these parameters could significantly affect the overall effectiveness of the drying process and quality of dried product [12,13].

### 2.3. Effect of Different Drying Procedures on the Quality of Gelatin

**Figure 1:**
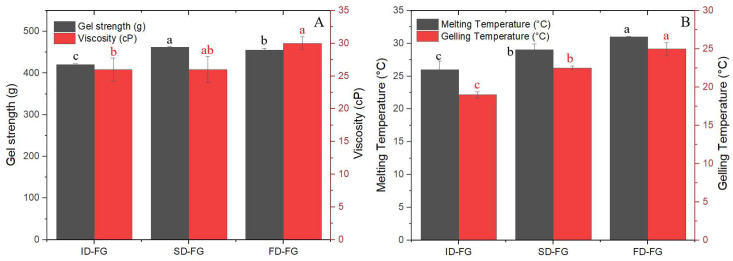
Quality and rheological properties of gelatin samples after various drying procedures. (**A**) Gel strength and viscosity; (**B**) gelling and melting temperatures. Means with different letters (a–c) within the same-colored bar are significantly different at *p* < 0.05.

**Figure 2:**
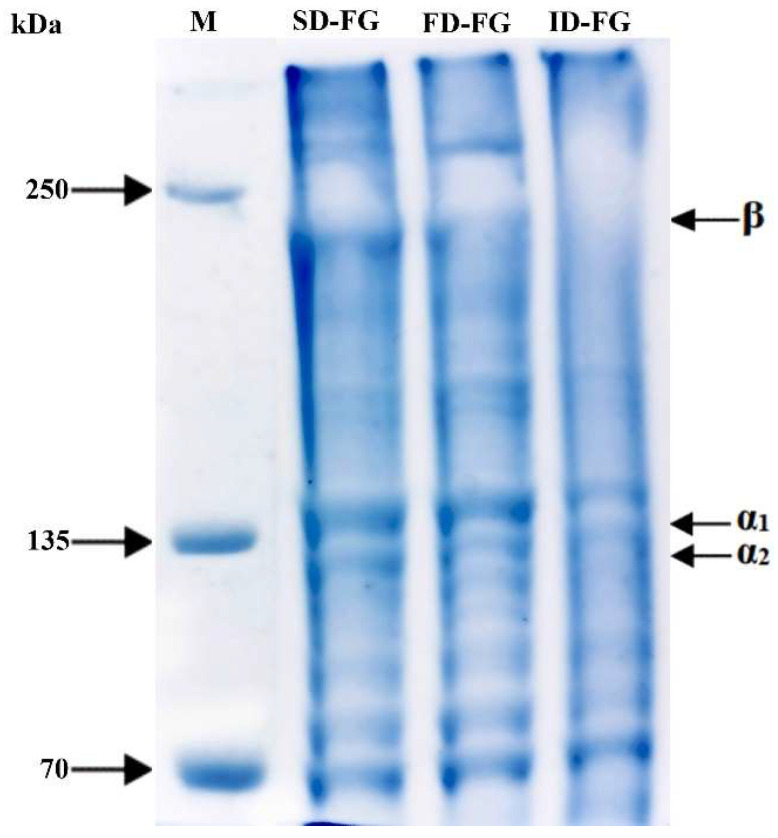
SDS pattern of gelatins after various drying procedures.

Gel strength and viscosity are crucial properties for assessing the quality of gelatins, which are influenced by the amino acid composition and the content of β- and γ-components. FD-FG and SD-FG demonstrated slightly higher gel strength compared to ID-FG (Figure 1). Mad-Ali et al. [26] reported that powdered gelatin with spray-drying at 160 °C exhibited gelling ability comparable to freeze-dried gelatin. Additionally, gel strength exhibits a strong correlation with the degree of hydrolysis, which influences the average molecular weight [19]. The reduced gel strength of the ID-FG sample was likely attributed to prolonged exposure time and thermal degradation during the drying process. Indeed, this observation was further supported by the molecular distribution profile (Figure 2), which indicated a slight decrease in the beta fractions in the ID-FG sample. Viscosity values, which were considered as another quality parameter of gelatin, were 24, 26 and 30 cP for ID-FG, SD-FG and FD-FG, respectively. Ahmad et al. [27] noted that a reduced content of high molecular weight chains (α- and β-) correlates with lower viscosity. However, it is widely recognized that changes in the number of cross-links and the balance of hydrogen bonds in solution also influence gelatin viscosity [28]. Considering the results, infrared drying exhibited gel strength and viscosity equivalent to those of the other drying methods.

The melting and gelling temperatures of dried gelatins are showed Figure 1B. The results indicated that both the gelling and melting temperature of FD-FG was significantly higher than those of ID-FG and SD-FG, while the difference between melting and gelling temperatures was almost the same. It is clear that freeze-drying, without thermal treatment, better preserves the structure of gelatin chains. This preservation leads to being more organized during the transitions between gelation and melting. Thus, the drying methods used for fish gelatin strongly affected the thermo-stability gel properties. Additionally, there exists a correlation between the melting point and molecular weight of gelatin; gelatins with lower molecular weights melt at lower temperatures compared to those with higher molecular weights [16,29].

### 2.4. Effect of Different Drying Procedures on Molecular Weight Distribution of Gelatin

The electrophoretic protein pattern (SDS Page) of fish skin gelatin obtained by the different methods is shown in Figure 2. The findings indicated that different drying methods exert distinct influences on the molecular structure of gelatins. ID-FG, SD-FG and FD-FG samples exhibited molecular patterns similar to type I collagen, characterized by two α_1_ and one α_2_ chain as the major components. However, the intensities of these chains varied depending on the drying process. Among all the samples, the FD-FG sample exhibited clearer bands in the characteristic regions of gelatin. Tkaczewska et al. [30] stated that lyophilized gelatin from carp (*Cyprinus carpio*) skin produced distinct and blurred bands. Although the molecular patterns of SD-FG gelatin were identical to FD-FG, it showed less intense bands in the α region. Spray-dried gelatin at 160 °C and 180 °C from carp exhibited patterns comparable to those of freeze-dried gelatin [26,30]. The most pronounced difference in protein profiles among the samples was observed in ID-FG (Figure 2). Although β bands were detected in all powdered gelatins, their presence was less distinct in the ID-FG sample. The β bands of the ID-FG could be affected by drying temperature and time, leading to possible hydrolysis of β fractions during dryings. Lower molecular weight proteins were observed in all samples, as clear bands, especially in FD-FG and SD-FG. However, this indicates a slight hydrolysis of gelatin proteins and explains the higher gel strength of the gelatin from spray- and freeze-drying [27].

### 2.5. Effect of Different Drying Procedures on Thermal Behavior of Gelatin

**Figure 3:**
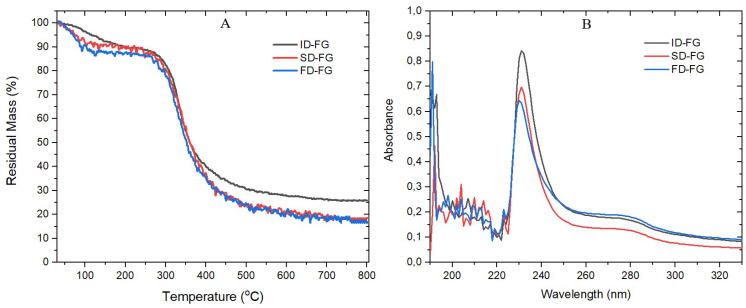
Thermal (**A**) and UV spectrum (**B**) profile of gelatin samples after various drying procedures.

Figure 3A illustrates the thermal decomposition characteristics of gelatins dried using infrared spray- and freeze-drying methods. The gelatins exhibited an identical behavior with regard to their decomposition stages. However, the TGA curves clearly indicated that the thermal decomposition behavior of gelatins varied against temperature. The first effect of temperature on the gelatin powders was attributed to the evaporation of physically absorbed water that was detected from 30 to 130 °C. The reason for this initial mass loss was the dehydration of water in the gelatin powder [31]. After the initial weight loss, the gelatin chains began to degrade, leading to a significant decrease in weight until approximately 430 °C. The weight loss was 64, 69, and 69% for ID-FG, SD-FG and FD-FG, respectively. However, during the later period, it was clearly seen that the ID-FG sample remained resilient and exhibited less weight loss. ID-FG powders at a temperature of 800 °C exhibited 29.60% residual weight, while the values for SD-FG and FD-FG powders were 17.20 and 15.45%, respectively. ID-FG gelatin powders demonstrated superior thermal stability, exhibiting greater thermal resistance compared to SD-FG and FD-FG. This could be attributed to the formation of strong hydrogen bonds and newly formed covalent bonds due to intermolecular and intramolecular protein interactions during the infrared drying process [32]. The findings suggested that infrared drying may be advantageous for gelatin with enhanced thermal stability compared to spray- and freeze-drying, particularly across diverse industrial applications.

### 2.6. Effect of Different Drying Procedures on UV–Vis Absorption Spectra of Gelatin

The UV-vis absorption spectra of powdered gelatins, dried using various methods, are presented in Figure 3B. The results demonstrate that ID-FG, SD-FG and FD-FG exhibit higher absorption in the wavelength range of 230–245 nm, indicating the presence of peptide bonds within the polypeptide chains of gelatin molecules [33]. The maximum absorption peaks of ID-FG, SD-FG and FD-FG were observed at 231, 231 and 230 nm, respectively. Commercial gelatins show the highest peak in the visible region, around 230 nm. As the degree of hydrolysis increases, there is an observed shift of the maximum absorption peaks towards longer wavelengths, and that gelatin with a broad molecular weight distribution exhibits higher maximum absorption peaks [2,33]. Among the gelatins, ID-FG showed the largest amplitude. This was likely related to the amide bond released as a result of partial hydrolysis during infrared drying. In fact, in Figure 2, partial hydrolysis confirmed, in molecular distribution, that β components could not be clearly determined in the ID-FG gelatin. All the gelatins showed minimal absorption in the 270–280 nm region, which could be attributed to the presence of non-aromatic residues such as phenylalanine, tyrosine, and tryptophan [33].

### 2.7. Effect of Different Drying Procedures on Surface Morphology of Gelatin

**Figure 4:**
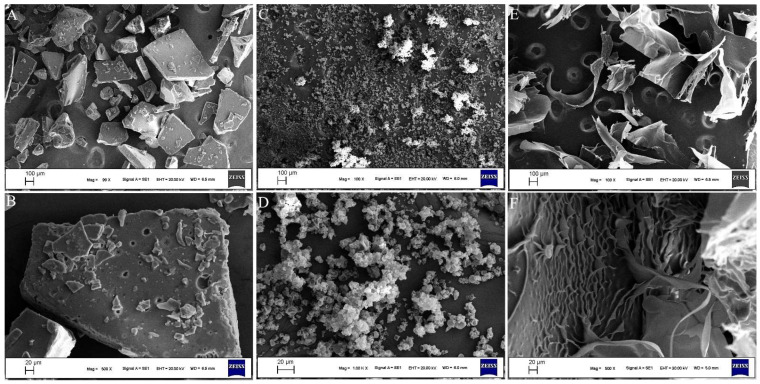
SEM visuals of gelatin samples after various drying procedures. ((**A**,**B**): gelatin powder obtained by ID; (**C**,**D**): gelatin powder obtained by SD; (**E**,**F**): gelatin powder obtained by FD).

SEM morphology of the gelatin obtained by different drying techniques is presented in Figure 4. SD-FG gelatins demonstrated a more homogeneous particle size distribution in contrast to FD-FG and ID-FG gelatins (Figure 4). The surface of the SD-FD powdered gelatin appeared folded or wrinkled, with particles exhibiting a smooth yet indented surface texture. The collapsed spheres may result from the asymmetric shrinkage of particles composed of gelatin and water. To enhance the rate of moisture evaporation, a drying droplet adjusts to minimize the diffusion path for water vapor, leading to the formation of a folded or wrinkled surface [22]. Under high magnification, FD-FG gelatin particles exhibited a sheet-like structure characterized by a crumpled, multi-layered formation. During the freezing of fish gelatin extracts, ice crystals nucleate from water solidification and intertwine with gelatin molecules. Following the conversion of these ice crystals, FD-FG exhibited a fragmented, glass-like, uneven sheet structure [34]. Thus, the formation of cavities resulting from the phase transition of ice crystals could also be observed. Consequently, FD-FG gelatin powders exhibited poor flowability and high cohesiveness, with higher Carr index and Hausner ratios compared to the other powdered samples (Table 1). ID-FG exhibited a dense and compact block structure, likely attributed to the separation of gelatin and water during the heating and drying processes. As the gelatin dissolved, it gradually precipitated at the bottom of the drying container, resulting in the formation of a sheet-shaped film [22,35]. In addition, this powder structure exhibited the best fluidity properties (Table 1).

## 3. Conclusions

Different drying methods were assessed for their impact on the quality and functional properties of the resulting fish skin gelatins. Results indicated that ID led to the lowest energy consumption and the shortest drying time. Also, gelatins dried by this method exhibited excellent quality, either superior in some of the quality parameters or with negligible or no significant difference from that of other two drying methods. The ID procedure exhibited similar gel strength (298 g) and viscosity (24 cP) compared to gelatins powdered by FD and SD methods. Furthermore, WHC, transparency, Carr index, Hausner ratio and solubility of ID-FG were higher than those of FD-FG. Based on the results, it is concluded that ID may significantly contribute to the thermal stability of powdered gelatin. Overall, it can be concluded that the ID procedure may be used for gelatin production with lower energy consumption, at lower cost and for similar gelatin products, if not superior. Based on the results presented in this study, further research may be conducted for a thorough analysis of the production cost for gelatin manufacturing by different drying methods. Furthermore, the results presented may be used by the gelatin industry for similar or superior product quality at lower cost in the future.

## 4. Materials and Methods

### 4.1. Materials

Fish skin from common carp (*C. luteus*) were obtained from local fish markets in Sanliurfa province. Upon transfer to the laboratory, the skins were washed and cleaned by removing scales and any residual flesh using a knife. Subsequently, the skins were maintained at −30 °C till use for gelatin extraction. All chemicals employed in the process were of analytical grade.

### 4.2. Methods

#### 4.2.1. Pretreatment and Gelatin Extraction

Gelatins were extracted from fish skins using the method described by Cansu [4]. Briefly, whole fish skins were lyophilized using a freeze dryer (Armfeld Limited-FT33, Ringwood, Hampshire, UK) for approximately 24 h at an absolute pressure of 20 mbar and a condenser temperature of −50 °C. Subsequently, the skins were cut into 3 × 3 cm pieces and immersed in a basic solution at a ratio of 1/20 (*w*/*v*) of 0.1 M NaOH for 1 h. Following this, the skins were rinsed with tap water and a paper towel was used to remove excess water. Then, skin samples were immersed in an acid solution at a ratio of 1/20 (*w*/*v*) of 0.1 M HCl for 1 h, and, afterwards, rinsed by tap water again. After these pretreatments for removal of residual fat and non-collagen proteins, 50 g of skin samples were immersed in 250 mL of deionized water for gelatin extraction at 55 °C for 4 h using a water bath and were shaken every 15 min by hand [4].

#### 4.2.2. Drying Processes

The gelatin extract obtained at the end of the extraction process exhibited a protein concentration of 0.186 mg/mL. The drying yields were calculated by dividing the weight of the obtained powdered gelatin (g) by the protein content (g) of the gelatin extract.

##### Infrared Drying (ID) of Gelatin Extract

Infrared-drying (ID) experiments were conducted with 375 W halogen infrared lamp, vertically placed at a distance of 15 cm from the extract, (Osram Sıccatherm, aY3, München, Germany) that was equipped with control unit (Ohaus, 6010H, Zurich, CH, USA). For infrared drying, 100 mL of gelatin extract was placed in a 12 cm diameter glass pan. As a result of preliminary trials, the drying process was carried out at 200 W [24]. The sample was distributed evenly and homogeneously over the entire glass pan, so that infrared radiation could be more effectively absorbed. Drying was continued until gelatin leaves were obtained, which was then referred as ID-FG. The resulting gelatin leaves were powdered with a blender and stored in sealed plastic bags at 4 °C till analyses were carried out.

##### Spray-Drying (SD) of Gelatin Extract

The raw gelatin extract was dried using a laboratory-scale spray dryer that was equipped with a nozzle atomizer with a diameter of 0.5 mm (Lab-Plant SD-04, Lab-Plant Ltd., Huddersfield, West Yorkshire, UK). A fixed extraction volume of 200 mL was used for all samples for the drying process, which was conducted under the following conditions: air inlet temperature: 160 °C feeding rate; 4 mL/min. During the drying, the outlet temperature was 88 ± 3 °C [7]. The powdered gelatins were stored in sealed plastic bags at 4 °C until analyses and was referred as SD-FG.

##### Freeze-Drying (FD) of Gelatin Extract

100 mL of gelatin extracts were transferred into plastic dishes with a diameter of 30 cm and subjected to freezing at −30 °C for 18 h. Subsequently, the frozen gelatin extracts were dried using a freeze-dryer (Armfield Limited-FT33, Ringwood, Hampshire, UK), which was operated at a condenser temperature of −50 °C and a vacuum pressure of 20 mbar for approximately 24 h. The resulting gelatin leaves were powdered by blender and were stored in airtight plastic bags at 4 °C until analyses were performed, and was referred to as FD-FG [7].

#### 4.2.3. Electricity and Energy Consumption Values of Drying Systems

Electricity consumption of the devices used for drying process was measured using a wattmeter (Luna, EN50470, Izmir, Türkiye). Prior to initiating the devices, the initial indexes on the wattmeter were recorded and upon completion of the drying process, the final indexes were also recorded. The total electricity consumption was determined by calculating the difference between the initial and the final indexes. The Specific Energy Consumption (SEC) (kWh/kg) was calculated by the given Equation (1), where P_ı_: the ratio of the total energy input (kW*h), and m_w_: the amount of water (kg) removed from the gelatin extract [21].

Specific energy consumption (SEC) (kW*h/kg) = P_1_/m_w_ (1)

#### 4.2.4. Solubility, Color, pH, Conductivity, Carr Index and Hausner Ratio of Dried Gelatins

The solubility of gelatins was detected according to the method described by Feng et al. [6], with minor adjustments. Gelatin solutions at a concentration of 1% (*w*/*v*) were dissolved at 60 °C and subsequently centrifuged at 6500 rpm. The amount of protein in the supernatant was determined using the Biuret method. Consequently, protein solubility was calculated by proportioning it to the total protein amount of the gelatin sample. Color values of gelatin powder samples were performed with the PCE-CSM 4 (PCE Instruments, Hochsauerland, Germany). A hybrid multimeter, Hanna, HI-2020 (Hanna instruments, Leighton Buzzard, UK), was employed to measure the pH and conductivity of samples at a 1% (*w*/*v*) concentration. The transparency of the gelatin solution was determined with a spectrophotometer (UVMini 1240 UV-VIS, Shimadzu, Kyoto, Japan) at a wavelength of 640 nm, as outlined by Cho et al. [36]. Carr index and Hausner ratio of powdered gelatins were determined using the method specified by Turchiuli et al. [37].

#### 4.2.5. Water-Holding and Fat-Binding Capacity

One gram of the powdered gelatin was weighed into pre-weighed 50 mL falcon tubes and mixed with 20 mL of pure water for assessing water-holding capacity (WHC), and with 20 mL of plant-based oil for evaluating fat-binding capacity (FBC). The mixtures were maintained at room temperature and vortexed at 15 s intervals for 15 min. Subsequently, centrifugation was performed at 6500 rpm for 10 min. The obtained supernatant was filtered through filter paper. WHC was calculated by weight gain, attributed to water retention, and expressed as grams of water per gram of gelatin (g water/g gelatin). Similarly, FBA was calculated by weight gain, attributed to fat retention, and expressed as grams of fat per gram of gelatin (g fat/g gelatin) [4].

#### 4.2.6. Gel Strength, Viscosity, Gelling and Melting Temperature

Gel strength analysis of the dried gelatins was conducted following the British Standard 757:1975 method [38]. Gel strength was measured using a TA-XT II Texture Analyzer (TA-XT II, Stable Micro Systems Ltd., Godalming, UK) fitted with a 12.7 mm diameter probe (P/0.5). A penetration depth of 4 mm was used for all measurements. Viscosity measurements were conducted on a 6.67% (*w*/*v*) gelatin solution following the standardized method outlined by GMIA [39]. A Cannon-Fenske routine calibrated viscometer was employed for all viscosity measurements.

A rotational rheometer (RV III Ultra, Brookfield, MA, USA) equipped with a low viscosity adaptor and a cylindrical spindle was used for the for gelling and melting temperature measurements. Cooling the sample was from 40 to 10 °C or heating from 10 to 40 °C at 10 s intervals and viscosity was recorded. The temperatures at which a sharp increase or decrease in viscosity was observed were recorded as the gelling and melting temperatures, respectively [40].

#### 4.2.7. SDS-Page Pattern of Powdered Gelatins

Sodium dodecyl sulfate–polyacrylamide gel electrophoresis (SDS-Page) was carried out to find the molecular weight profile of gelatins [41]. In brief, the powders were dissolved in 5% SDS at 60 °C and denatured at 90 °C for 5 min in a water bath. Samples were adjusted to final concentration of 2 mg/mL by adding 2 folds of sample buffer (2% SDS, 0.002% bromophenol blue, 5% mercaptoethanol). Following the application, 15 μL of each sample was applied onto a polyacrylamide gel composed of 5% stacking and 10% separating gels, and electrophoresis was conducted under a constant voltage of 120 V. After electrophoresis, the gels were stored in a gel fix solution consisting of 10% glacial acetic acid and 50% methanol for 45 min. Subsequently, they were transferred to the dyeing solution (G20 Stain, Bio-rad, Hercules, CA, USA) and kept for 45 min. Finally, the gels were destained using a solution containing 50% methanol and 7.5% glacial acetic acid.

#### 4.2.8. Thermal Analysis

The thermogravimetric profile of the gelatin powders was examined using a thermogravimetric analyzer (Shimadzu DTG-60H, Shimadzu Corporation, Kyoto, Japan) in platinum TGA pans. Heating was conducted at a rate of 10 °C/min within the temperature limits of 30 to 800 °C under an inert (N2) atmosphere [42].

#### 4.2.9. UV-Vis Absorption Spectrum

UV absorption spectra of powdered gelatins were analyzed using a modified version of the method outlined by Ruan et al. [2]. For the analysis, 25 mg of gelatin powders were solubilized in 50 mL of 0.5 M acetic acid solution and maintained in a 60 °C water bath for 30 min. The spectrum profile was determined within the 190–330 nm range at a scanning speed of 0.2 nm/s using a blank-corrected spectrophotometer (UV-Mini 1240 UV-VIS, Shimadzu, Kyoto, Japan) [2].

#### 4.2.10. Morphological Characterization of Gelatin Powders

Microstructure and surface morphology of gelatin powders were examined by the Scanning electron microscopy (SEM) (Zeiss Sigma 300, Zeiss Co., Oberkochen, Germany). Prior to image acquisition, gelatin powders were frozen and then coated with gold. The samples were imaged at a magnification of 3.00 and 5.00 K × and an acceleration voltage of 10 kV [9].

### 4.3. Statistical Analysis

All experimental analyses were conducted at least in triplicate and mean values were given with ±standard deviation. Datasets were subjected to one-way ANOVA and subsequently compared using Tukey’s HSD test at a 95% significance level. SPSS v22.0 (Chicago, IL, USA) was used for statistical analysis.

## Figures and Tables

**Table 1:** Physical features of gelatin samples after various drying procedures.

	ID-FG	SD-FG	FD-FG
WHC (g water/g protein)	5.37 ± 0.7 ^b^	10.22 ± 1.1 ^a^	2.11 ± 0.1 ^c^
FBA (g oil/g protein)	0.75 ± 0.0 ^c^	2.10 ± 0.1 ^b^	15.39 ± 0.2 ^a^
Solubility (%)	96.36 ± 3.6 ^a^	96.55 ± 2.5 ^a^	90.15 ± 3.1 ^b^
Hausner ratio	1.34 ± 0.03 ^c^	1.56 ± 0.05 ^b^	1.73 ± 0.03 ^a^
Carr index	25.26 ± 1.50 ^c^	35.94 ± 2.21 ^b^	42.09 ± 1.10 ^a^
pH	3.67 ± 0.0 ^a^	3.68 ± 0.0 ^a^	3.62 ± 0.0 ^a^
Transparency	39.10 ± 0.5 ^b^	53.00 ± 0.4 ^a^	26.80 ± 0.1 ^c^
Conductivity (µS/cm)	670 ± 5.0 ^a^	670 ± 4.5 ^a^	693 ± 6.8 ^a^
L*	70.90 ± 2.2 ^c^	95.40 ± 1.0 ^a^	80.47 ± 1.2 ^b^
a*	6.10 ± 0.5 ^a^	3.55 ± 0.3 ^b^	−0.75 ± 0.2 ^c^
b*	30.70 ± 0.8 ^a^	7.32 ± 0.7 ^c^	14.85 ± 1.1 ^b^
Hue angle	1.38 ± 0.0 ^a^	1.12 ± 0.0 ^b^	−1.51 ± 0.0 ^c^
Chroma	31.32 ± 0.9 ^a^	8.15 ± 0.6 ^c^	14.90 ± 1.0 ^b^

Means with different letters (a–c) within the same row are significantly different at *p* < 0.05. ID-FG; infrared-dried fish gelatin, SD-FG; spray-dried fish gelatin, FD-FG; freeze-dried fish gelatin, WHC; water holding capacity, FBA; fat binding ability.

**Table 2:** Electricity and productivity value of gelatin samples after various drying procedures.

	ID-FG	SD-FG	FD-FG
Electricity consumption (kW*h/g gelatin)	0.217 ± 0.00 ^c^	1.784 ± 0.01 ^a^	1.528 ± 0.03 ^b^
Specific Energy Consumption (SEC) (kW*h/kg)	3.41 ± 0.10 ^c^	8.46 ± 0.31 ^b^	25.33 ± 0.24 ^a^
Drying time (min/g gelatin)	45.96 ± 0.11 ^c^	64.18 ± 0.04 ^b^	147.16 ± 1.3 ^a^
Drying yield (%)	94.70 ± 0.13 ^b^	60.31 ± 0.51 ^c^	96.32 ± 0.20 ^a^

Means with different letters (a–c) within the same row are significantly different at *p* < 0.05. ID-FG; infrared-dried fish gelatin, SD-FG; spray-dried fish gelatin, FD-FG; freeze-dried fish gelatin.

## Data Availability

Data will be made available on request.
